# Supplementary figures and images for: Pharmacological rescue of impaired mitophagy in Parkinson’s disease-related LRRK2 G2019S knock-in mice
Source: eLife. 2021 Aug 3;10:e67604. doi: 10.7554/eLife.67604 (PMC8331189; doi:10.7554/eLife.67604)

LRRK2

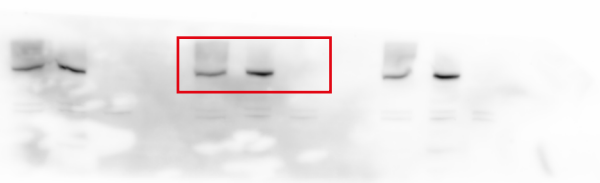

Tubulin

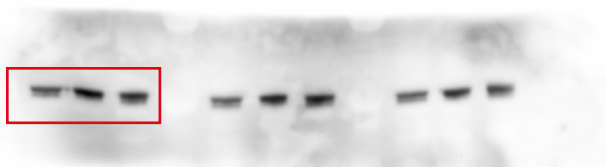

Supplement: Figure 1—source data 2. [file elife-67604-fig1-data2.zip › Figure 1-source data 2 - C/Figure 1-source data 2 - C.pdf]

LRRK2

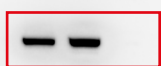

Tubulin

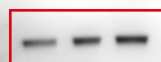

Supplement: Figure 1—source data 3. [file elife-67604-fig1-data3.zip › Figure 1-source data 3 - F/Figure 1-source data 3 - F.pdf]

pS935 LRRK2

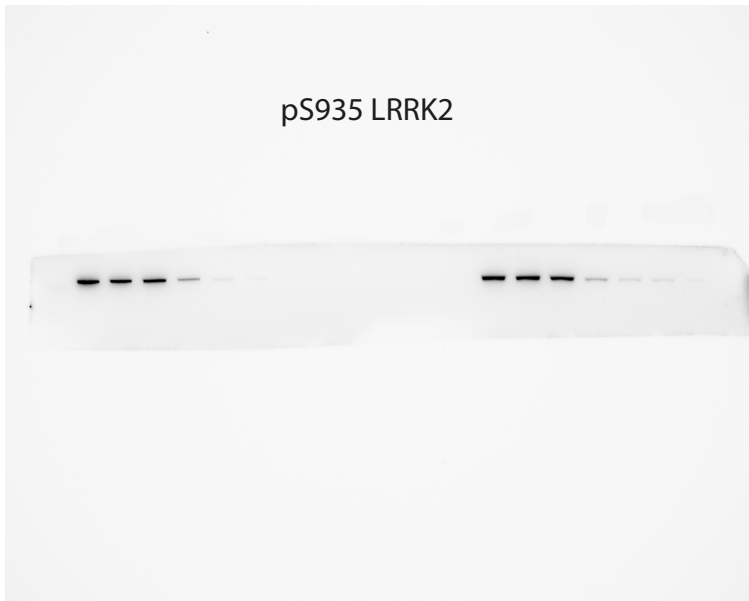

LRRK2

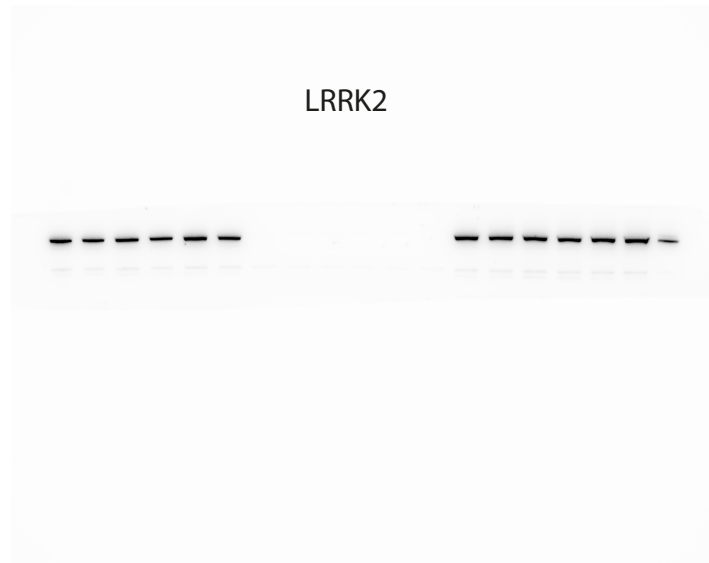

pT73 Rab10

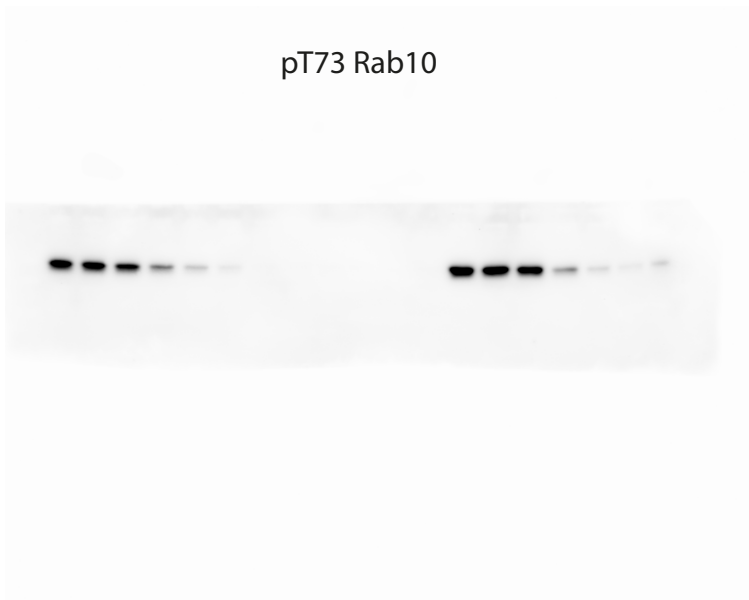

Rab10

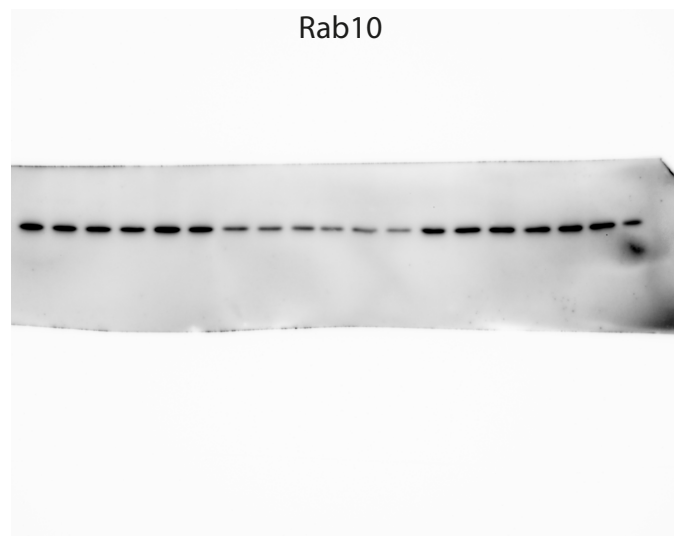

Tubulin

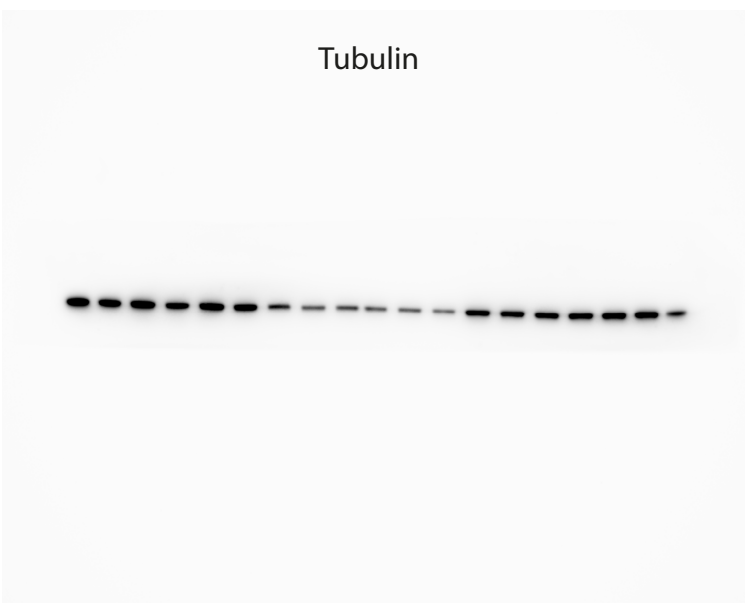

Supplement: Figure 1—source data 4. [file elife-67604-fig1-data4.zip › Figure 1-source data 4 - H/Figure 1-source data 4 - H.pdf]

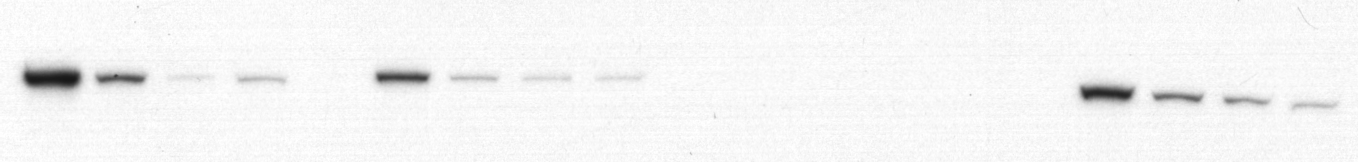

Supplement: Figure 1—figure supplement 1—source data 2. [file elife-67604-fig1-figsupp1-data2.zip › Figure 1-figure supplement 1-source data 2 - E/Mefs dose response UDD2 15.12..17.tiff]

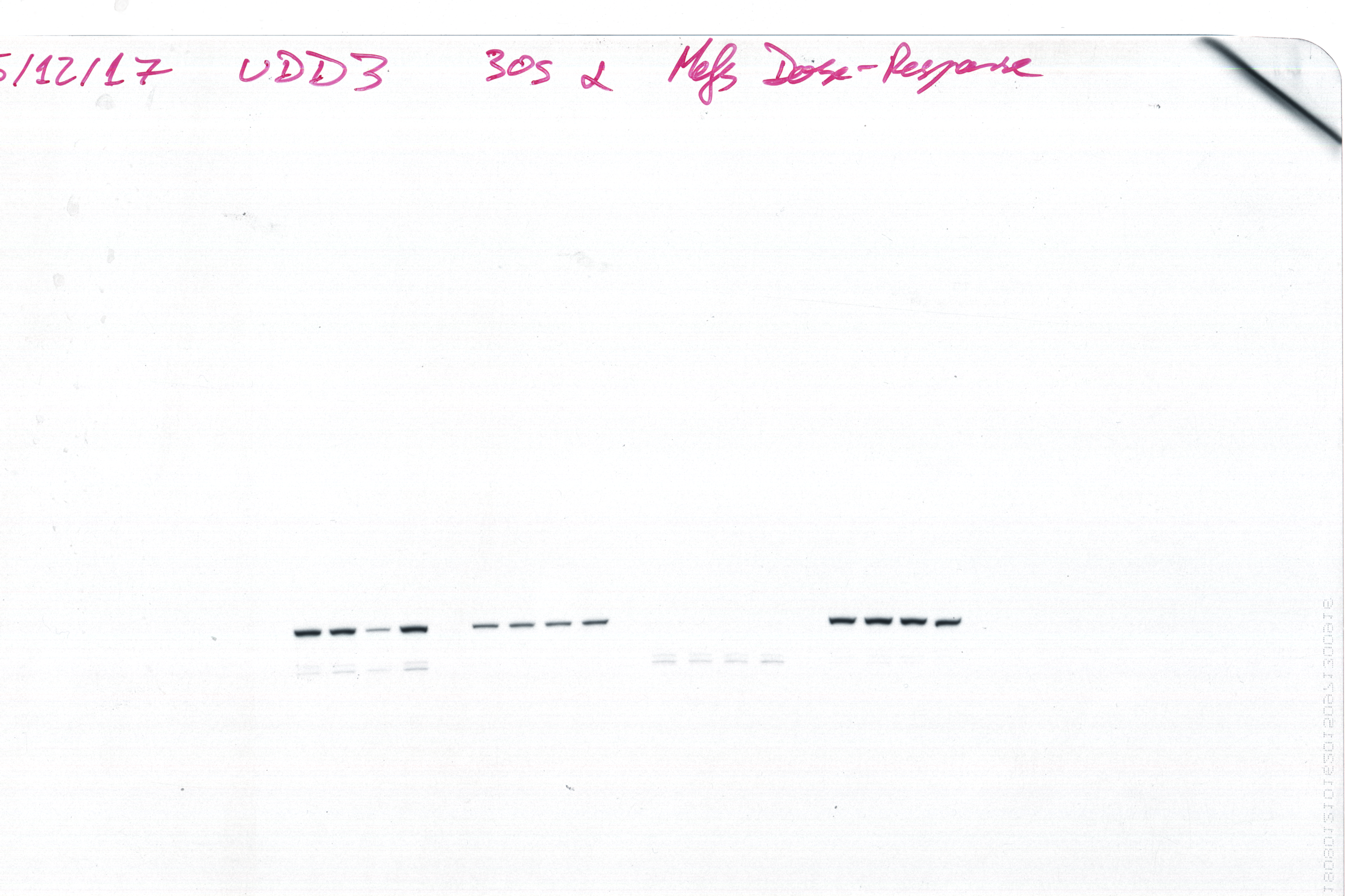

Supplement: Figure 1—figure supplement 1—source data 2. [file elife-67604-fig1-figsupp1-data2.zip › Figure 1-figure supplement 1-source data 2 - E/Total LRRK2 MLi-2.tiff]

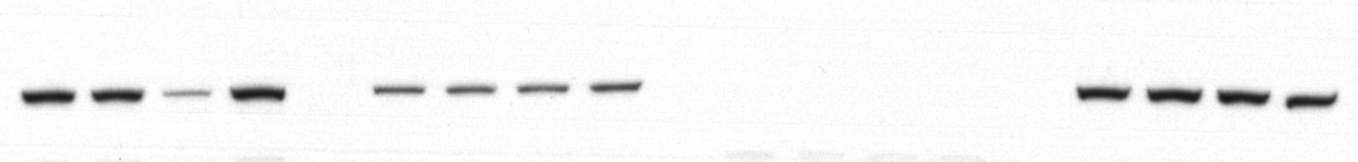

Supplement: Figure 1—figure supplement 1—source data 2. [file elife-67604-fig1-figsupp1-data2.zip › Figure 1-figure supplement 1-source data 2 - E/Mefs dose response UDD3 15.12..17.tiff]

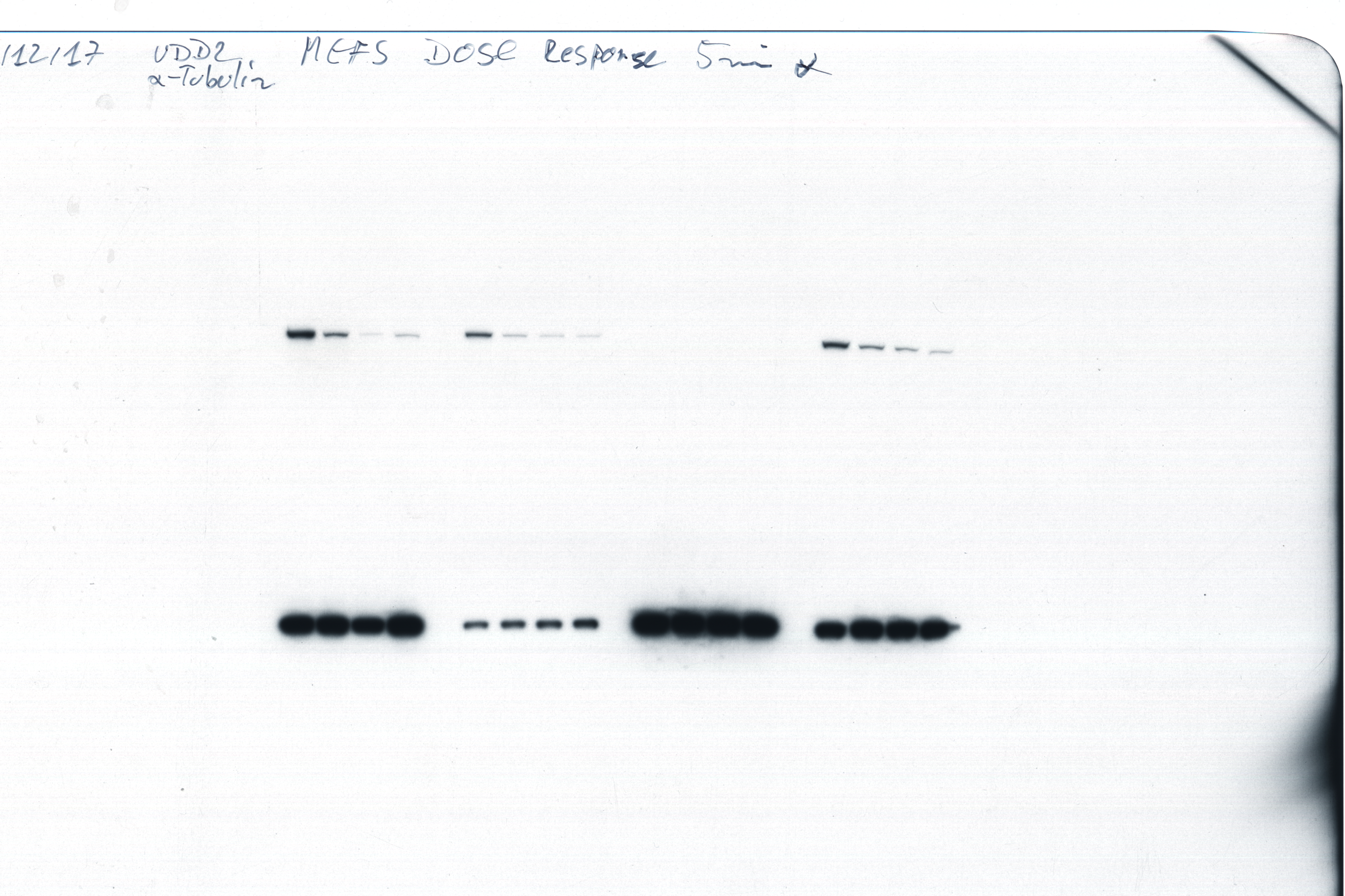

Supplement: Figure 1—figure supplement 1—source data 2. [file elife-67604-fig1-figsupp1-data2.zip › Figure 1-figure supplement 1-source data 2 - E/pS935 LRRK2 MLi-2.tiff]

pS65 Ubiquitin

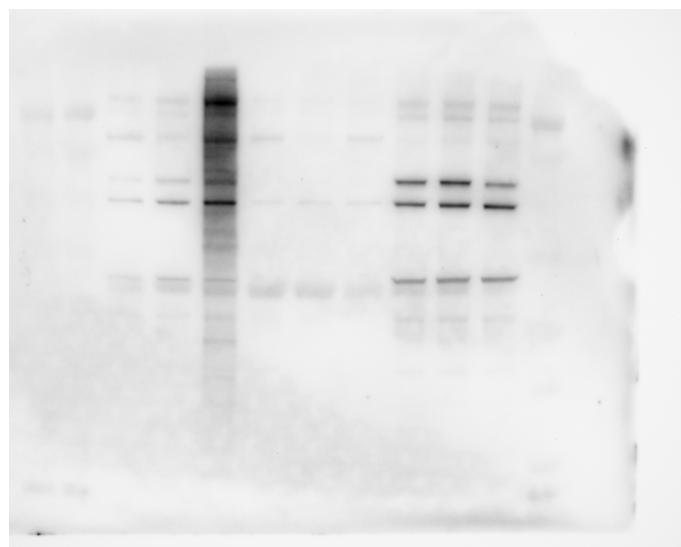

Total Ubiquitin

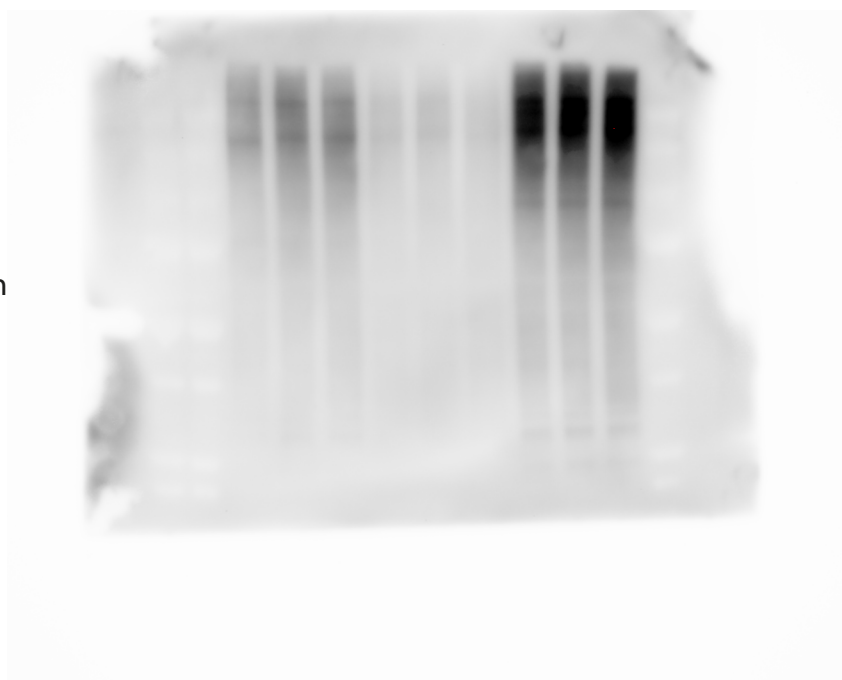

Ponceau

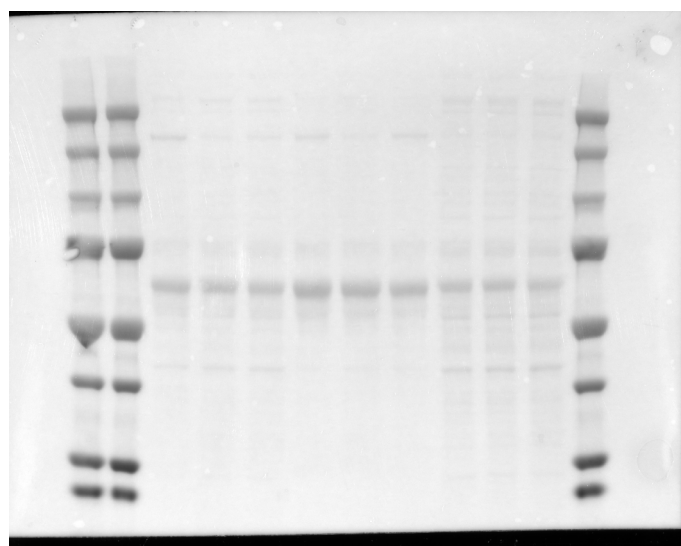

Supplement: Figure 2—source data 2. [file elife-67604-fig2-data2.zip › Figure 2-source data 2 - I/Figure 2-source data 2 - I.pdf]

pS935 LRRK2

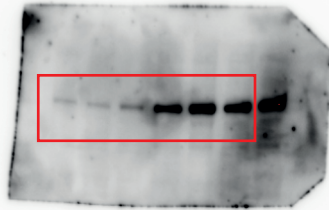

LRRK2

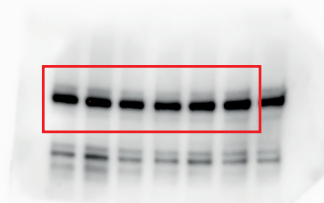

Supplement: Figure 2—figure supplement 1—source data 2. [file elife-67604-fig2-figsupp1-data2.zip › Figure 2-figure supplement 1-source data 2/Figure 2-figure supplement 1-source data2-A.pdf]

Parkin

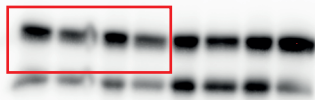

phospho-Ubiquitin

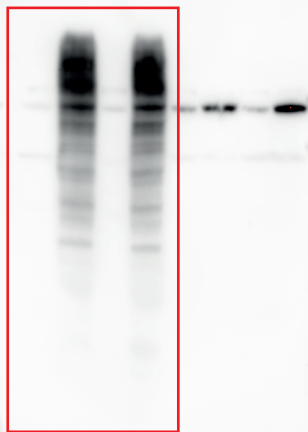

Supplement: Figure 2—figure supplement 1—source data 3. [file elife-67604-fig2-figsupp1-data3.zip › Figure 2-figure supplement 1-source data 3/Figure 2-figure supplement 1-source data 3-F.pdf]

pS935 LRRK2

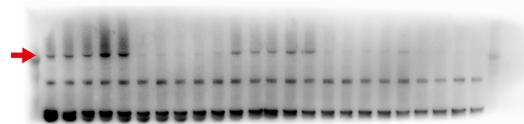

pS106 Rab 12

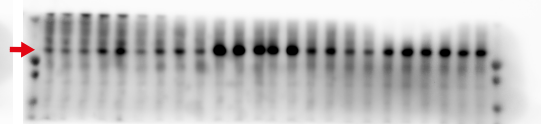

HSP60

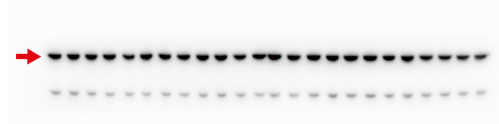

LRRK2

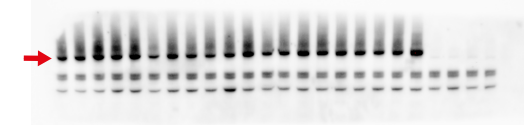

Rab12

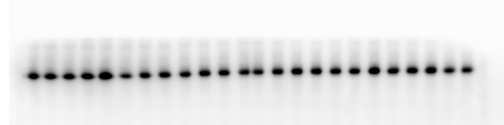

TOMM20

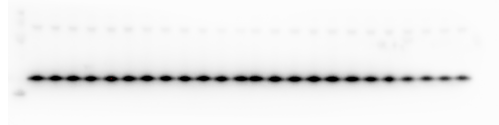

PGC-1a

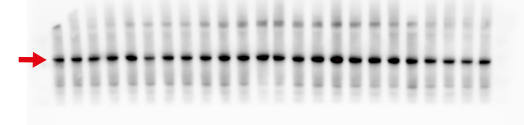

PGC-1B

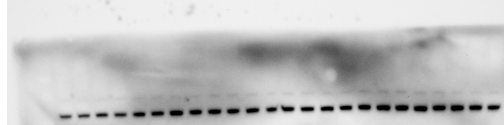

TFAm

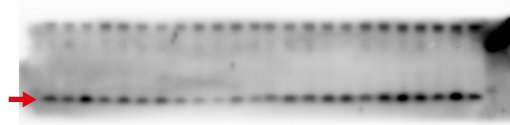

p62

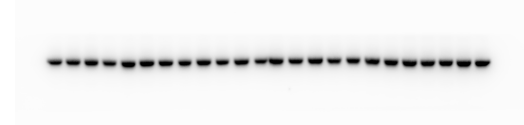

LC3

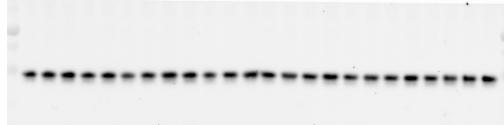

Actin

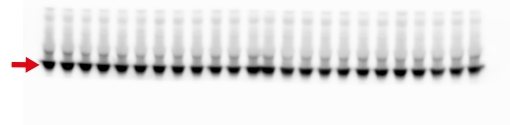

Supplement: Figure 5—source data 2. [file elife-67604-fig5-data2.zip › Figure 5-source data 2/Figure 5-source data 2 - A.pdf]

Kidney

pS935 LRRK2

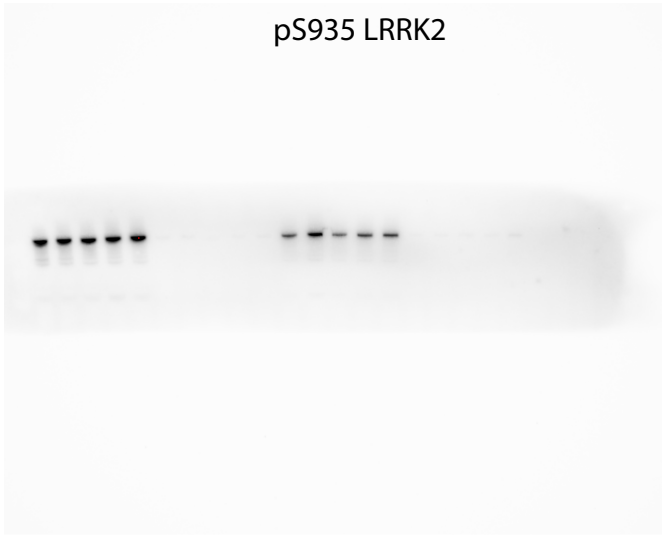

pT73 Rab10

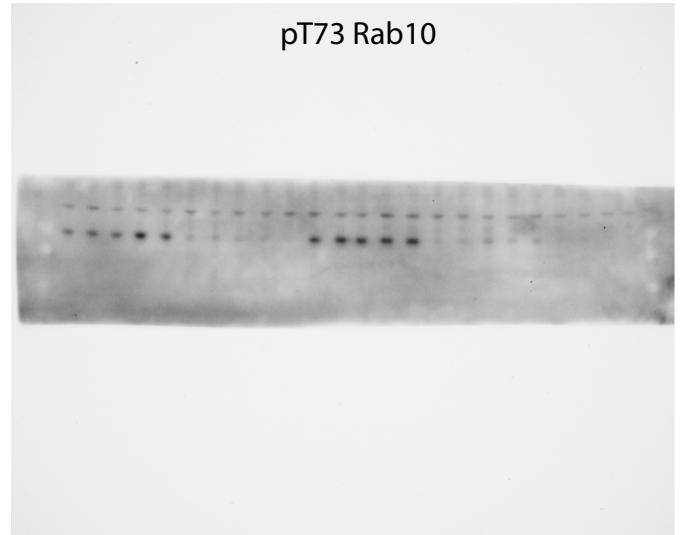

LRRK2

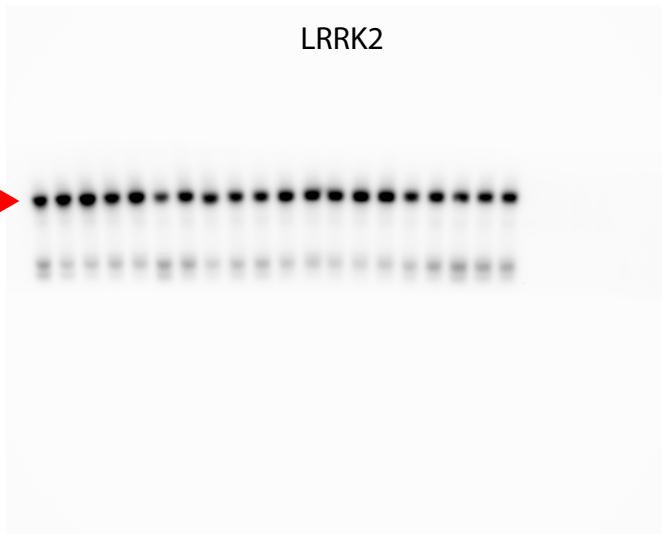

Rab10

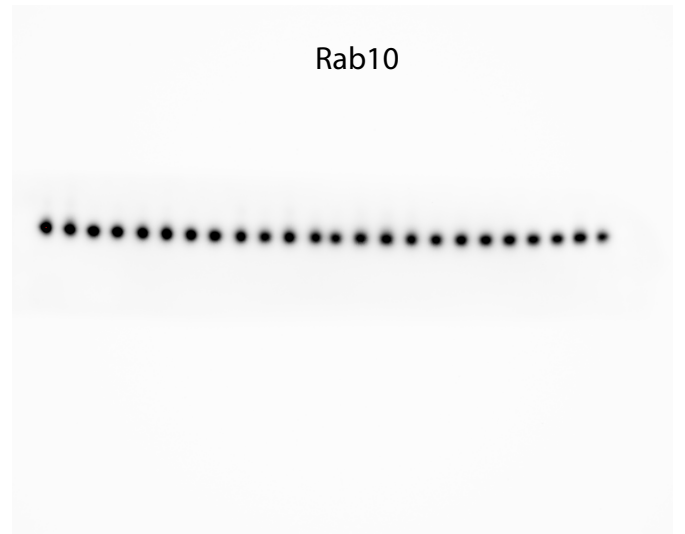

Actin

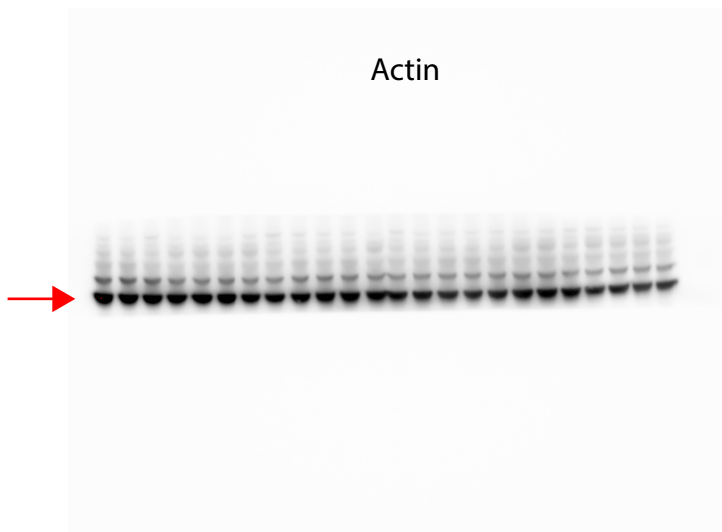

Supplement: Figure 5—figure supplement 1—source data 2. [file elife-67604-fig5-figsupp1-data2.zip › Figure 5-figure supplement 1-source data 2/Figure 5-figure supplement 1-source data 2 - Kidney.pdf]

Lungs

pS935 LRRK2

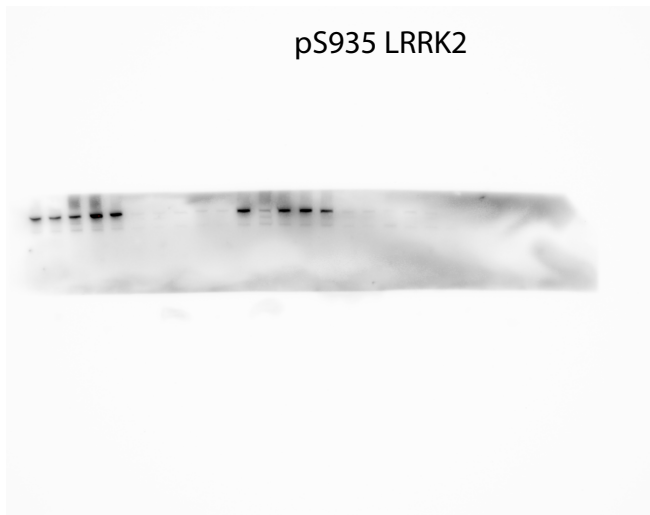

pT73 Rab10

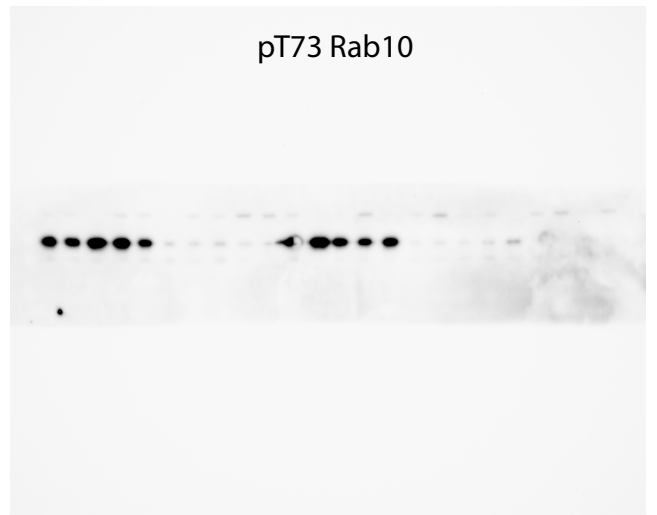

LRRK2

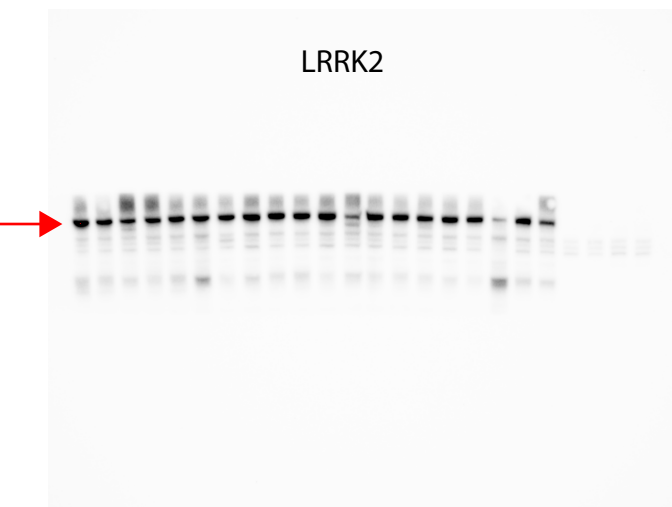

Rab10

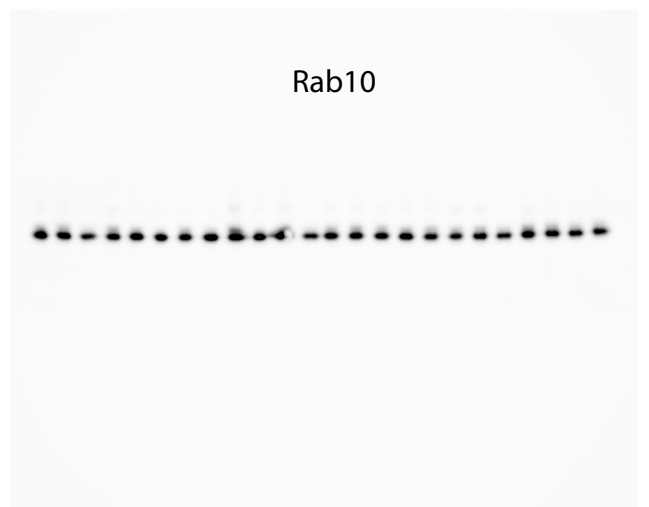

Actin

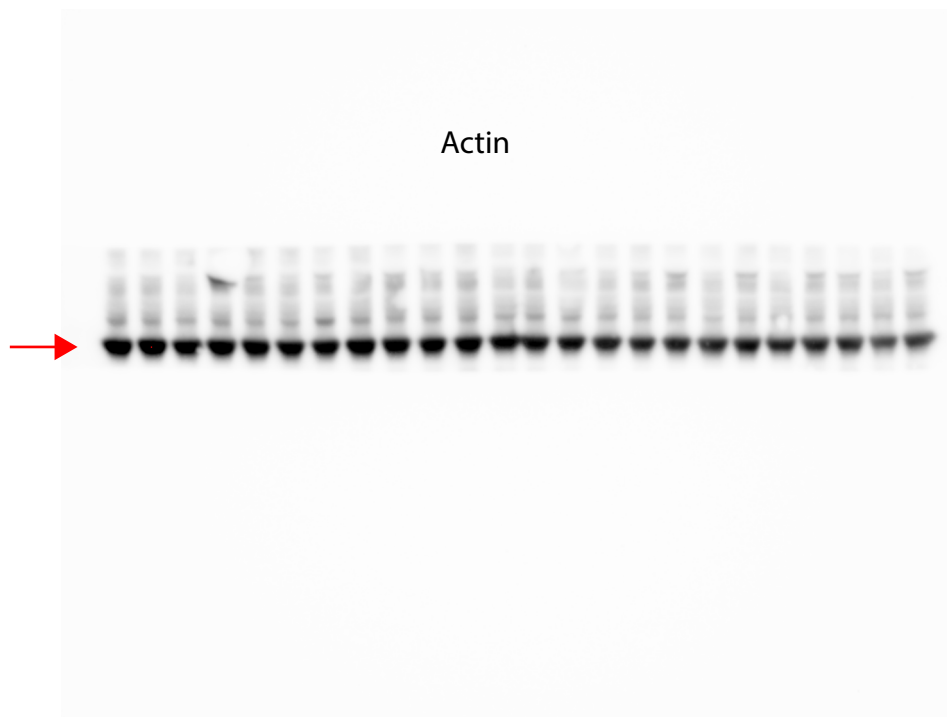

Supplement: Figure 5—figure supplement 1—source data 2. [file elife-67604-fig5-figsupp1-data2.zip › Figure 5-figure supplement 1-source data 2/Figure 5-figure supplement 1-source data 2 - Lungs.pdf]
