# Supplementary material for: Pharmacological rescue of impaired mitophagy in Parkinson’s disease-related LRRK2 G2019S knock-in mice
Source: eLife. 2021 Aug 3;10:e67604. doi: 10.7554/eLife.67604 (PMC8331189; doi:10.7554/eLife.67604)

GSK2578215A

MLi-2

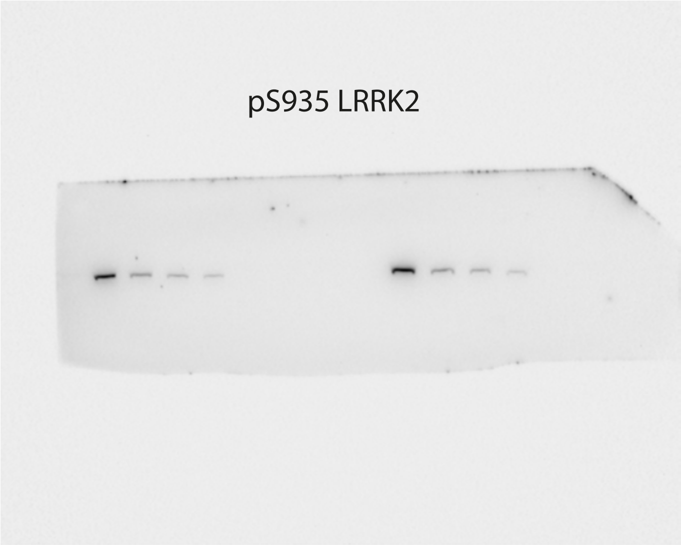

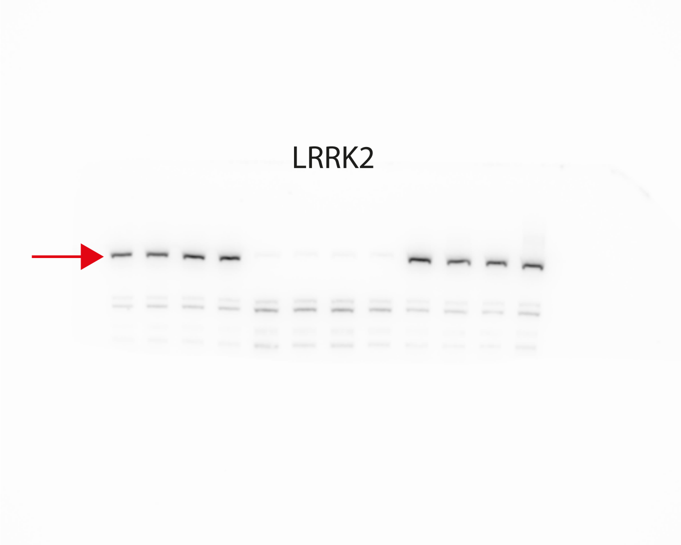

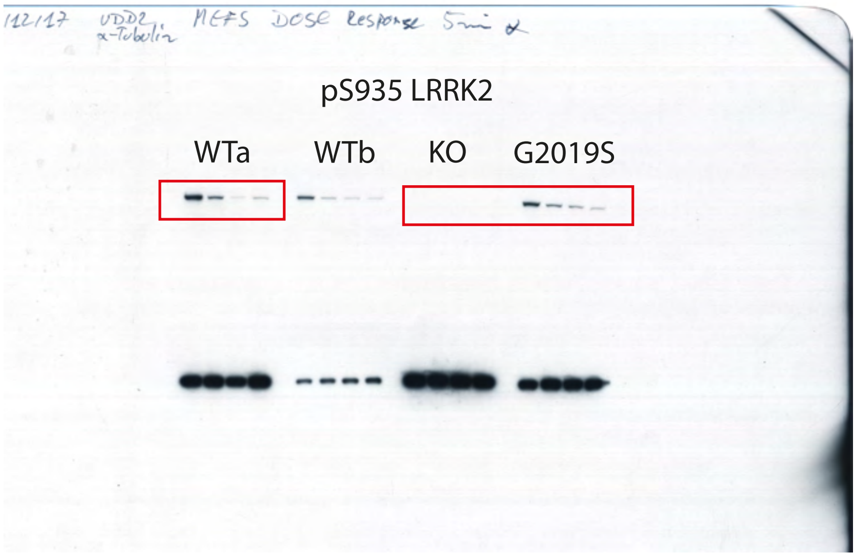

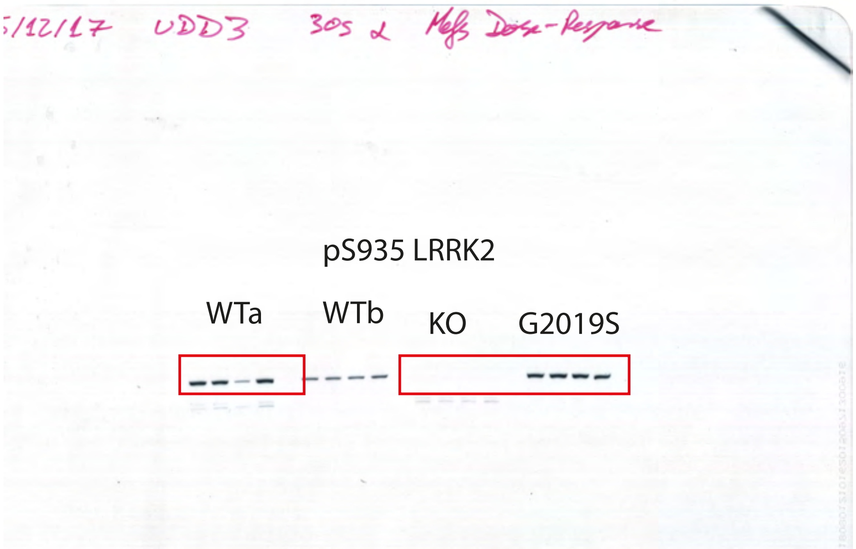

Supplement: Figure 1—figure supplement 1—source data 2. [file elife-67604-fig1-figsupp1-data2.zip › Figure 1-figure supplement 1-source data 2 - E/Figure 1-figure supplement 1-source data 2 - E.pdf]
